# Supplementary material for: ZNF703 mRNA-Targeting Antisense Oligonucleotide Blocks Cell Proliferation and Induces Apoptosis in Breast Cancer Cell Lines
Source: Pharmaceutics. 2023 Jul 11;15(7):1930. doi: 10.3390/pharmaceutics15071930 (PMC10384502; doi:10.3390/pharmaceutics15071930)
Supplement: Supplementary file 1 [file pharmaceutics-15-01930-s001.zip › pharmaceutics-2452008-supplementary.pdf]

Supplementary Figures

Figure S1: ASO design for ZNF703 mRNA targeting and selection ASOs by WB analysis.

A

Names	Position	N	Seq_5.3	Antisens_3.5	Antisens_5_3	GC	Specificity
ASO_2	250	20	ATCTAACCCAAGGACACCCG	TAGATTGGGTTCCTGTGGGC	CGGGTGTCCTTGGGTTAGAT	55%	100%
ASO_9	390	20	AGATGCTGAGCGCTCACACC	TCTACGACTCGCGAGTGTGG	GGTGTGAGCGCTCAGCATCT	60%	100%
ASO_13	470	20	GAGCTGGACGCCAAGAAGAG	CTCGACCTGCGGTTCTTCTC	CTCTTCTTGGCGTCCAGCTC	60%	100%
ASO_17	550	20	CTCCAAACTCAACTCGGTGG	GAGGTTTGAGTTGAGCCACC	CCACCGAGTTGAGTTTGGAG	55%	100%
ASO_24	690	20	TCAAGCCCTACTCCAAGGGC	AGTTCGGGATGAGGTTCCCG	GCCCTTGGAGTAGGGCTTGA	60%	100%
ASO_26	730	20	CAAAGACAGCGGCTCCTCCT	GTTTCTGTCGCCGAGGAGGA	AGGAGGAGCCGCTGTCTTTG	60%	100%
ASO_37	950	20	GAGCTGGACAAGAAAGACCA	CTCGACCTGTTCTTTCTGGT	TGGTCTTTCTTGTCCAGCTC	50%	100%
ASO_51	1230	20	TGGATCCTAGCAAGTCCGGC	ACCTAGGATCGTTCAGGCCG	GCCGGACTTGCTAGGATCCA	60%	100%
ASO_57	1350	20	GATTATGCCGCGACCCCTAT	CTAATACGGCGCTGGGGATA	ATAGGGGTCGCGGCATAATC	55%	100%
ASO_67	1550	20	CCGCTCTACACCTACGGCTT	GGCGAGATGTGGATGCCGAA	AAGCCGTAGGTGTAGAGCGG	60%	100%
ASO_68	1570	20	CATGCTGCAGAACGAACCGC	GTACGACGTCTTGCTTGGCG	GCGGTTCGTTCTGCAGCATG	60%	100%
ASO_81	1830	20	TGCGGAATCCACACACTTTG	ACGCCTTAGGTGTGTGAAAC	CAAAGTGTGTGGATTCCGCA	50%	100%
ASO_86	1930	20	CGGACCCTACTATTCGCCAT	GCCTGGGATGATAAGCGGTA	ATGGCGAATAGTAGGGTCCG	55%	100%
ASO_87	1950	20	ACGCGCTGTATGGACAGAGA	TGCGCGACATACCTGTCTCT	TCTCTGTCCATACAGCGCGT	55%	100%

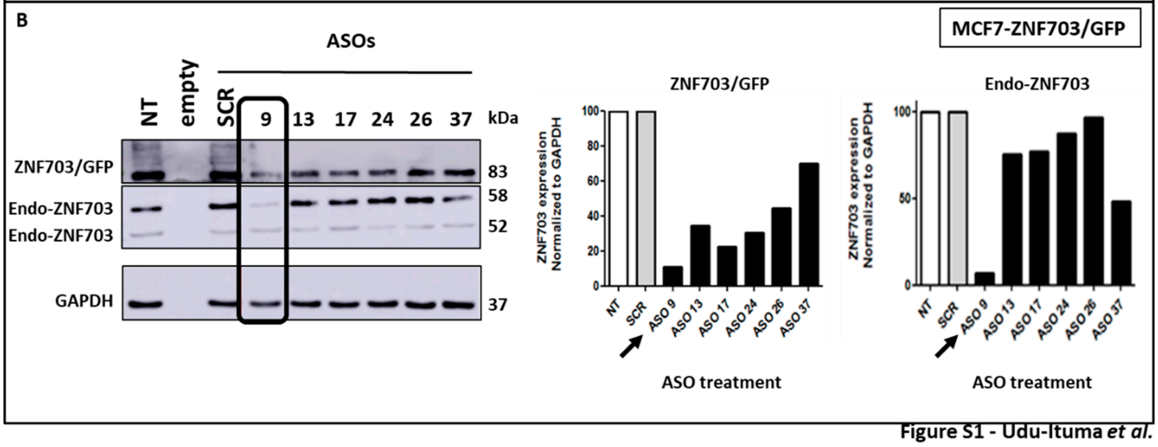

Figure S1 - Udu-Ituma *et al.*

Legend of Figure S1: ASO design for ZNF703 mRNA targeting and selection ASOs by WB analysis. The table lists 14 ASO designed for *ZNF703* mRNA targeting as well as their characteristics (**A**). Protein lysates of MCF7-ZNF703/GFP BC cell line treated with 100nM of each of them (here ASO9, 13, 17, 24, 26, 37) or control-ASO (scrambled, SCR) are analysed to assess ZNF703 expression by WB (**B**). WB images showing ZNF703 band signals intensities were respectively quantified for ZNF703/GFP (83kDa) and endo-ZNF703 (58kDa) by Image J, normalized to GAPDH and represented as bars. ASO9 was clearly identified to show higher downregulation efficiency and effectively represses both the endogenous and exogenous *ZNF703* mRNA.

Figure S2: IC50 calculation for ASO9 in five breast cancer cells.

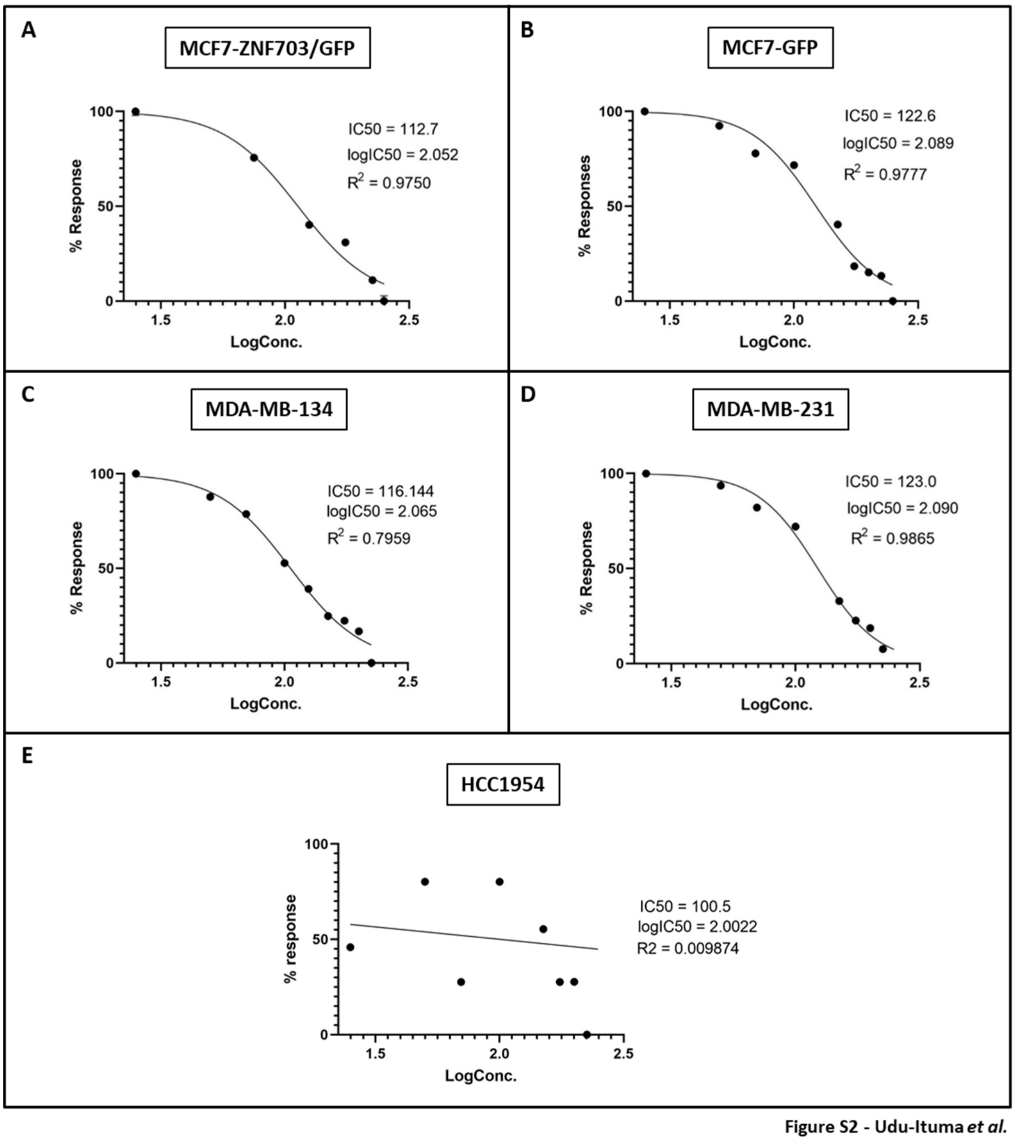

Legend of Figure S2: IC50 calculation for ASO9 in five breast cancer cells. IC50 calculation for ASO9 was done in MCF7-ZNF703/GFP, MCF7-GFP, MDA-MB 134, MDA-MB 231 and HCC1954 breast cancer cells. Each experiment was done in triplicate. MCF7-ZNF703/GFP exhibited the most sensitive response to ASO9 (112.7 nM). IC50 values 122.6, 116.1 and 123 nM were established for MCF7-GFP, MDA-MB-134 and MDA-MB-231 (**A-D**), respectively. HCC1954 cell line was used as a negative control (**E**)

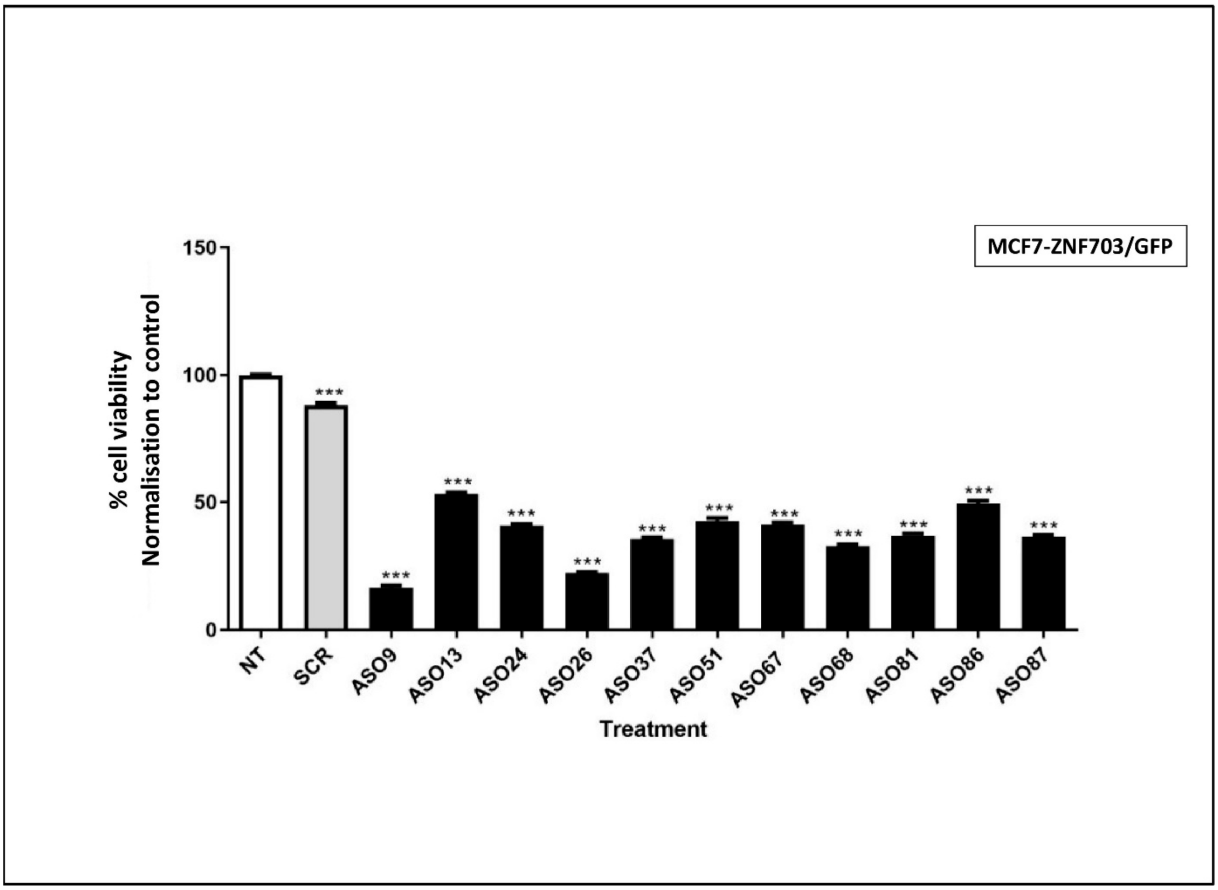

Legend of Figure S3. Cell viability tests on MCF7-ZNF703/GFP after treatment with 11 various ASOs against *ZNF703* mRNA. Cell viability assessment on MCF7-ZNF703/GFP cells was measured with MTT assay after treatment with 11 various ZNF703-ASOs (200 nM) against *ZNF703* mRNA and compared with SCR. This evaluation was done in triplicate. SCR exhibited weak toxicity. All ZNF703-ASO induces a significant decrease in cell viability (Student's t-test, *** $p \leq 0.001$). ZNF703-ASO9 was the most efficient ASO. SCR treatment exhibited only weak toxicity.
